# The Association of Preoperative PET-CT and Survival in Patients with Resectable Cervical Cancer

**DOI:** 10.3390/jcm11237143

**Published:** 2022-12-01

**Authors:** Chih-Hsiung Su, Wan-Ming Chen, Ming-Chih Chen, Ben-Chang Shia, Szu-Yuan Wu

**Affiliations:** 1 Department of Accounting Information, Chihlee University of Technology, Taipei 220305, Taiwan; 2 Graduate Institute of Business Administration, College of Management, Fu Jen Catholic University, Taipei 24205, Taiwan; 3 Artificial Intelligence Development Center, Fu Jen Catholic University, Taipei 24205, Taiwan; 4 Department of Food Nutrition and Health Biotechnology, College of Medical and Health Science, Asia University, Taichung 41354, Taiwan; 5 Division of Radiation Oncology, Lo-Hsu Medical Foundation, Lotung Poh-Ai Hospital, Yilan County 265010, Taiwan; 6 Big Data Center, Lo-Hsu Medical Foundation, Lotung Poh-Ai Hospital, Yilan County 265010, Taiwan; 7 Department of Healthcare Administration, College of Medical and Health Science, Asia University, Taichung 41354, Taiwan; 8 Cancer Center, Lo-Hsu Medical Foundation, Lotung Poh-Ai Hospital, Yilan County 265010, Taiwan; 9 Centers for Regional Anesthesia and Pain Medicine, Taipei Municipal Wan Fang Hospital, Taipei Medical University, Taipei 11031, Taiwan; 10 Department of Management, College of Management, Fo Guang University, Yilan County 262307, Taiwan

**Keywords:** preoperative ^18^FDG-PET–CT, cervical cancer, surgery, survival, stages

## Abstract

Purpose: No randomized study with a long-term follow-up has investigated the effect of pretreatment 18-fluorodeoxyglucose positron emission tomography–computed tomography (^18^FDG-PET–CT) on the survival of patients with stage IB-IIA cervical cancer receiving curative surgery. Therefore, in this propensity score–matched, population-based cohort study, we investigated the effect of preoperative ^18^FDG-PET–CT on the survival outcomes of patients with potentially resectable cervical cancer. Patients and Methods: We included 2550 patients with stage IB-IIA cervical cancer receiving curative surgery with complete data on clinical stages. The patients were categorized into two 1:4 propensity, score–matched groups depending on whether they underwent pretreatment ^18^FDG-PET–CT, and their outcomes were compared. Results: We included 2030 and 520 patients with cervical cancer in the non-pretreatment and pretreatment PET–CT groups, respectively. Multivariable analyses revealed that the most prominent correlation between preoperative PET–CT and all-cause death was observed in the patients with stage IB–IIA cervical cancer receiving surgery (aHR [95% CI]: 1.16 [0.83–1.63]; *p* = 0.3752). Conclusions: Preoperative ^18^FDG-PET–CT was not associated with longer survival in the patients with clinical stage IB–IIA cervical cancer receiving curative surgery.

## 1. Introduction

In Taiwan, cervical cancer is the ninth most common cancer among women [1]. In 2020, cervical cancer accounted for an estimated 604,000 new cancer cases and 342,000 deaths worldwide [2], and was the fourth most common cancer among women [3]. For most patients with early-stage IB–IIA cervical cancer, curative surgery is suggested rather than primary radiotherapy (RT). Primary RT with or without chemotherapy is recommended for patients who are not candidates for primary surgery due to medical comorbidities, poor functional status, or limited health resources [4,5,6]. Compared with surgery, primary RT is more likely associated with higher long-term morbidity [4,5,6]. Surgery for cervical cancer is a valuable curative-intent treatment strategy [7,8], irrespective the administration of neoadjuvant or adjuvant treatment.

The National Comprehensive Cancer Network (NCCN) guidelines suggest performing preoperative 18-F fluorodeoxyglucose (^18^FDG)-positron emission tomography (PET)–computed tomography (CT) (^18^FDG-PET–CT) in patients with cervical cancer to define the disease extent and evaluate pelvic and para-aortic lymph nodes [9]. However, the cost-effectiveness of preoperative ^18^FDG-PET–CT for patients with cervical cancer remains debatable [10,11,12], especially in early stages. The discrepancy between the findings of previous studies [10,11,12] and the recommendation of the NCCN [9] has raised a concern regarding the clinical value of preoperative ^18^FDG-PET–CT for cervical-cancer staging and its effect on the survival outcomes of patients with cervical cancer. Thus, whether preoperative ^18^FDG-PET–CT should be performed in patients with potentially resectable cervical cancer, especially in areas with limited medical resources remains unclear.

We conducted this long-term, nationwide, head-to-head, propensity scored–matched (PSM) study to investigate the effect of preoperative ^18^FDG-PET–CT on the survival outcomes of patients with potentially resectable cervical cancer. The findings of this study can help oncologists to make clinical decisions and policymakers to establish health policies regarding the use of valuable medical equipment.

## 2. Patients and Methods

### 2.1. Study Design and Patient Data Source

This retrospective study was conducted using data from the Health and Welfare Data Center (HWDC) established by Taiwan’s Ministry of Health and Welfare. The HWDC consolidates data gathered by the Taiwanese government from various sources. These data are then de-identified and made available for research purposes based on case-by-case approval. In particular, we used data from the Taiwan Cancer Registry, which includes the detailed staging and treatment information of patients with cancer; the Cause of Death database, which lists all death certificates issued in Taiwan [13]; and the National Health Insurance Research Database, which contains the billing information on all National Health Insurance (NHI)-reimbursed examinations, medications, and treatments. The NHI program has been implemented since 1995 and covers more than 99% of Taiwan’s population. Since July 2004, the NHI program has covered ^18^F-FDG–PET performed for the initial staging of cervical cancer when optimal staging could not be achieved through conventional CT. All databases in the HWDC are linked through a common but anonymized identifier to ensure privacy. The requirement for informed consent was waived due to the retrospective and de-identified nature of the study data.

### 2.2. Study Sample

We selected consecutive patients aged at least 20 years who were clinically confirmed to have cervical cancer between 1 January 2009 and 31 December 2018. We excluded patients with cancers before cervical cancer and those with cervical cancer with clinical stages IIB-IV from the analysis.

### 2.3. Covariates and Outcome Definition

We extracted data on age, years of diagnosis, Charlson comorbidity index (CCI), American Joint Committee on Cancer (AJCC) clinical stage, differentiation, histological type, medical center or not, AJCC clinical stage, surgical margin status, and neoadjuvant/adjuvant treatment at the last follow-up date from the Taiwan Cancer Registry. Clinical stages were based on the AJCC 8th edition for cervical cancer. Age was analyzed as a continuous variable. We selected patients with squamous cell carcinoma (SCC) or adenocarcinoma. All patients with clinical stage IB-IIA resectable cervical cancer underwent surgery and adjuvant treatments, such as chemotherapy, RT, or adjuvant CCRT. In our study, resectability was defined and verified by gynecologic oncologists and was performed in all the included patients with cervical cancer. Modified radical or radical hysterectomy was conducted as the standard surgery for cervical cancer. Modified radical hysterectomy includes the removal of the uterus, cervix, upper one-fourth of the vagina, and parametria. The index date was the date of surgery.

From the National Health Insurance Research Database (NHIRD), we identified patients who underwent ^18^F-FDG-PET–CT within 0 to 60 days before the index date. Patients with a record of ^18^F-FDG-PET–CT were considered to have undergone preoperative PET–CT, whereas those without records were considered to have not undergone preoperative PET–CT. For the non-PET group, clinical stages were determined on the basis of the findings of chest, abdominal, and pelvic CT and pelvic magnetic resonance imaging (MRI) with contrast. For the PET group, clinical stages were determined on the basis of the findings of chest, abdominal, and pelvic CT; pelvic MRI with contrast; and PET–CT. Nuclear- medicine physicians and radiologists officially reviewed and provided reports on all the images. All PET–CT reports were interpreted by nuclear-medicine physicians. The Taiwan Cancer Registry Database (TCRD) is unique because all PET–CT reports are reviewed and reported by well-trained nuclear-medicine physicians. Moreover, the Taiwan Cancer Registry Administration randomly reviews the reports of images through peer review to verify the accuracy of diagnoses, and hospitals with outlier chargers or practices may be audited and subsequently be heavily penalized if malpractice or discrepancies are identified. The primary outcome of interest was all-cause death, which was determined from the index date to the date of death. Information on overall survival (OS) was obtained from the Cause of Death database. Patients whose death records could not be found were considered alive, and their data were censored on the last day of the database record (31 December 2019).

### 2.4. Propensity Score Matching

After adjustment for confounders, Szu-Yuan Wu used a Cox-proportional-hazards model to model time from the index date to all-cause death for patients with cervical cancer who underwent surgery. In this study, we used propensity score matching (PSM) to reduce the effects of confounders and control for them and to examine the preoperative PET–CT directionality of the survival effect. Matching variables used were age, years of diagnosis, CCI score, AJCC clinical stage, differentiation, histological type, and medical center or nonmedical center. However, imbalance was still noted in some covariates, namely the postoperative factors AJCC clinical stage, surgical margin status, and neoadjuvant/adjuvant treatment [14]. For CCI score calculation, comorbidities were determined based on *International Classification of Diseases*, *Ninth Revision*, and *Clinical Modification* (*ICD-9-CM*) codes, or, *International Classification of Diseases*, *Tenth Revision*, and *Clinical Modification* (*ICD-10-CM*) codes in the main diagnosis in inpatient records or outpatient records if the number of outpatient visits was ≥2 within 1 year. Comorbidities with onset 12 months before the index date were recorded. We matched the cohorts at a ratio of 4:1 by using the greedy method, and covariates were matched using a propensity score within a caliper of 0.2 [15].

### 2.5. Statistical Analysis

Continuous data are presented as mean ± standard deviation or median and interquartile range, as applicable, whereas categorical data are presented as number and percentage. The distribution of patient characteristics was compared using the χ^2^ test for categorical variables and the independent *t* test or Kruskal–Wallis test for continuous variables.

Survival curves were generated using the Kaplan–Meier method and were compared using the log-rank test. Cox-proportional-hazards models were used to estimate the hazard ratio (HR) and 95% confidence interval (CI), and to determine the effects of covariates on OS. We examined the interaction effect by adding (clinical stages) × (predictor) in the univariable and multivariable models by using PROC PHREG (SAS version 9.4) and estimated the crude HR stratified by AJCC clinical stages based on the traditional concept of resectability and unresectability [16]. Table 1 lists the sample sizes for different AJCC stages. All statistical analyses were performed using SAS (version 9.4; SAS Institute Inc., Cary, NC, USA). A two-sided *p* value of <0.05 was considered statistically significant.

### 2.6. Study Registered on a National Database

The study protocols were reviewed and approved by the Institutional Review Board of Tzu-Chi Medical Foundation (IRB109-015-B).

## 3. Results

### 3.1. Patient Characteristics

After PSM, a final cohort of 520 patients (mean age: 51.27 ± 12.18 years) who underwent preoperative PET–CT and 2030 patients (mean age: 51.51 ± 12.66 years) who did not undergo preoperative PET–CT were included in this study. Most of the covariates were balanced between the case and control groups; however, more patients had advanced clinical stages and received neoadjuvant/adjuvant treatment in the preoperative PET–CT group than in the non-PET–CT group. Furthermore, more patients had surgical-margin positivity in the non-PET–CT group than in the PET–CT group. The median follow-up periods of the preoperative PET–CT and non-PET–CT groups were 5.26 and 5.89 years, respectively.

### 3.2. Predictors of Survival

The aHR (95% CI) of the prognostic factors of all-cause death, namely CCI ≥ 1 (1.37 [1.13–1.66]; *p* = 0.0015); clinical stages IIA (1.09 [1.04–1.50]; *p* < 0.0001); surgical margin positivity (1.98 [1.66–2.36]; *p* < 0.0001); and adenocarcinoma (1.85 [1.54–2.22]; *p* < 0.0001), were associated with poor OS in multivariate analysis (Table 2).

### 3.3. Consistency between Clinical Stage and Pathologic Stage

Table 3 shows the consistency between the clinical and pathologic stages determined through PET or non-PET methods for the propensity score-matched patients with cervical cancer receiving curative surgery. Higher consistency between the clinical and pathologic stages for the clinical stage IB-IIA was noted in the non-PET group. The accuracies were 65.93% versus 75.87% and 70.51% versus 83.09% for the clinical stages IB and IIA, respectively, between the preoperative PET–CT and non-PET–CT groups.

## 4. Discussion

No randomized controlled trial (RCT) including a large sample size and long-term follow-up has examined the effect of preoperative ^18^FDG-PET–CT on the survival outcomes of patients with cervical cancer, although the NCCN guidelines recommend performing ^18^FDG-PET–CT for patients with cervical cancer [9]. A retrospective comparative study reported that preoperative PET–CT exhibited low sensitivity and had a minimal clinical effect on the preoperative planning of stage IB–IIA cervical cancer [10]. Because of the discrepancy in the findings of previous studies, whether preoperative ^18^FDG-PET–CT should be performed in patients with potentially resectable cervical cancer remains unclear. Therefore, we conducted this head-to-head PSM study to investigate the diagnostic accuracy of preoperative ^18^FDG-PET–CT for patients with cervical cancer receiving curative surgery and the effect of ^18^FDG-PET–CT on the long-term survival outcomes of these patients. The risk of all-cause death associated with preoperative ^18^FDG-PET–CT was observed in the patients with stage IB–IIA cervical cancer receiving surgery (aHR [95% CI]: 1.16 [0.83–1.63]; *p* = 0.3752). The accuracy of preoperative ^18^FDG-PET–CT versus no ^18^FDG-PET–CT was 65.93% versus 75.87% and 70.51% versus 83.09% for the clinical stages IB and IIA, respectively (Table 3). The accuracy of staging was lower in the preoperative ^18^FDG-PET–CT group than in the non-^18^FDG-PET–CT group after surgery with pathologic proof for resectable-cervical-cancer stages. 

We determined CCI ≥ 1 [17,18]; advanced stages [18,19]; surgical-margin positivity [20,21]; and adenocarcinoma [18,22,23,24] as negative prognostic factors for OS in the patients with resectable cervical cancer receiving curative surgery; these findings are in agreement with those in previous studies [17,18,19,20,21,22,23,24]. Mortality was higher in the high CCI group with cervical cancer [17]. Surgical-margin positivity might result in a high risk of recurrence and might be associated with poor OS in the patients with cervical cancer receiving surgery [20,21]. This is the first study to demonstrate surgical-margin positivity as an independent risk factor for not only local recurrence but also all-cause mortality. Zhang et al. reported that, compared with curative surgery for cervical SCC, curative surgery for cervical adenocarcinoma was associated with poorer OS and higher metastatic rates [18].

**Figure 1:**
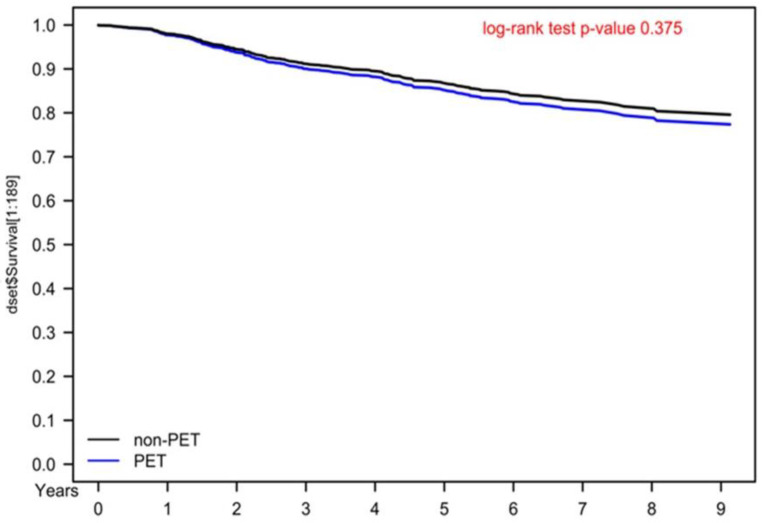
Kaplan–Meier overall survival curves for propensity score–matched cervical cancer patients receiving curative surgery.

No RCT has indicated that the use of preoperative PET–CT is superior to chest/abdominal/pelvic CT and pelvic MRI in terms of survival outcomes or diagnostic accuracy [10,11]. A retrospective study including 47 patients with cervical cancer receiving surgery examined the clinical validation of PET–CT and demonstrated that PET–CT had little value in patients with early-stage MRI-defined lymph node–negative cervical carcinoma [11]. Because the likelihood of metastatic-nodal disease is considerably low in women with early-stage cervical cancer, Driscoll et al. indicated that PET–CT should not be included in the routine-pretreatment evaluation of these women. However, a retrospective study including 87 patients with early-stage cervical cancer reported that PET–CT was valuable for detecting lymph-node metastases [12]. Taken together, the findings regarding the use of preoperative PET–CT for early-stage cervical cancer remain controversial; whether oncologists should perform preoperative PET–CT for patients with potentially resectable cervical cancer remains unclear, especially its benefits for OS. This is the first study to indicate that preoperative PET–CT exhibited no survival benefits in our PSM population with cervical cancer (Table 2 and Figure 1). Moreover, the more inaccurate staging of advanced cervical cancer (Table 3) was noted in the preoperative PET–CT group than in the non-PET–CT group. This is the first comparative study with a long-term follow-up period to evaluate the association between survival outcomes and preoperative PET–CT use in patients with resettable cervical cancer receiving surgery. Preoperative PET–CT for cervical cancer might not be necessary due to the relatively low sensitivity and specificity of PET–CT compared with those of chest/abdominal/pelvic CT and pelvic MRI.

Performing an RCT to evaluate the survival outcomes of patients with cervical cancer receiving and not receiving preoperative PET–CT is difficult because preoperative PET–CT is recommended by NCCN. Compelling the nonuse of preoperative PET–CT in an RCT for patients with cervical cancer might be an ethical problem. Striking a balance among confounders between patients with cervical cancer receiving and not receiving preoperative PET–CT (i.e., case and control groups, respectively)—a main requirement of the RCT design—is impossible [25]. Therefore, a PSM-based design, such as that used in the current study, can resolve this problem by maintaining a balance among confounders between the case and control groups without any bias. Moreover, PSM is currently the recommended standard tool for estimating the effects of covariates in studies where any potential bias exists [15,26]. Although the main advantage of PSM is that it enables the precise estimation of the covariate effect, PSM cannot control for factors not accounted for in the model. Moreover, PSM is predicated on an explicit selection bias of those who could be matched; in other words, individuals who could not be matched are not part of the scope of inference. Our study is the first to use an effective PSM design mimicking an RCT to investigate the effect of preoperative PET–CT on the survival outcomes of patients with cervical cancer receiving surgery. A large and well-designed RCT should be conducted in the future to confirm the effects of preoperative PET–CT on the survival outcomes of patients with stage IB-IIA cervical cancer receiving surgery, although the inclusion of a control arm (non-pretreatment PET–CT) for patients with cervical cancer may be an the ethical problem. Because of the difficulty in performing such an RCT, a large retrospective observational study might be necessary. However, when a large cohort requiring in-depth post-processing, such as PSM, is included, a retrospective study of an existing database without randomization cannot mimic an RCT, resulting in selection bias. Thus, the use of a well-designed PSM study can address the problem of using available data and complement the lack of a well-designed RCT.

The strength of our study is that, it is the first, largest, and long-term follow-up cohort study to include integrated PET–CT as the homogenous modality and to estimate the effect of preoperative PET–CT on the survival outcomes of patients with cervical cancer receiving surgery stratified by different clinical stages. No study comparing different clinical stages and including a sufficient sample size, a long-term follow-up period, ^18^FDG-PET–CT as the homogenous modality, and a well-designed PSM mimicking an RCT, has been performed. The results of the current study indicated that preoperative ^18^FDG-PET–CT was not associated with survival benefits in the patients with early-stage IB–IIA cervical cancer. Our results suggest that preoperative ^18^FDG-PET–CT is not necessary for patients with potentially resectable cervical cancer. Our findings should be considered in future clinical practice and prospective clinical trials. Our results would be valuable for policymakers to establish appropriate policies regarding the use of this expensive medical modality for patients with cancer.

## 5. Conclusions

Preoperative ^18^FDG-PET–CT was not associated with longer survival in the patients with clinical stage IB–IIA cervical cancer receiving curative surgery. Irrespective of the early or advance stage, the accuracy of staging was lower in the preoperative PET–CT group than in the non-PET–CT group after surgery with pathologic validation.

## Figures and Tables

**Table 1:** Clinicodemographic characteristics of patients with cervical cancer with and without pretreatment PET–CT scan before curative surgery (after propensity-score matching).

	No Preoperative PET-CT		Preoperative PET-CT		*p* Value
	N = 2030		N = 520		
	N	%	N	%	
**Age** (mean ± SD)	51.51 ± 12.66		51.27 ± 12.18		0.1451
**Age median** (IQR) (years)	51.00 (42.00–59.00)		51.50 (41.00–58.00)		0.0734
**Age groups** (years)					0.5650
Age ≤ 40	404	19.90%	116	22.31%	
40 < Age ≤ 50	490	24.14%	128	24.62%	
50 < Age ≤ 60	629	30.99%	157	30.19%	
Age > 60	507	24.98%	119	22.88%	
**Years of Diagnosis**					0.8731
2008–2010	499	24.58%	124	23.85%	
2011–2014	707	34.83%	177	34.04%	
2015–2018	824	40.59%	219	42.12%	
**CCI Scores**					0.1956
0	1718	84.63%	428	82.31%	
1	312	15.37%	92	17.69%	
**AJCC clinical stage**					0.9999
IB	1421	70.00%	364	70.00%	
IIA	609	30.00%	156	30.00%	
**Differentiation**					0.4160
I (well differentiated)	160	7.88%	47	9.04%	
II (moderately differentiated)	1105	54.43%	270	51.92%	
III (poorly differentiated)	694	34.19%	191	36.73%	
IV (undifferentiated)	71	3.50%	12	2.31%	
**Histological type**					0.6097
Adenocarcinoma	629	30.99%	172	33.08%	
Squamous cell carcinoma	1401	69.01%	348	66.92%	
**Medical Center**	2030		520		0.4115
No	1203	59.26%	296	56.92%	
Yes	827		224	0.4308	
**Surgical margin**					0.0004
Margin negative	1622	79.90%	451	86.73%	
Margin positive	408	20.10%	69	13.27%	
**Neoadjuvant or Adjuvant treatment**					<0.0001
Adjuvant CCRT	651	32.07%	217	41.73%	
Neoadjuvant chemotherapy	114	5.62%	48	9.23%	
Neoadjuvant CCRT	261	12.86%	68	13.08%	
No neoadjuvant/adjuvant treatments	754	37.14%	145	27.88%	
Adjuvant RT	250	12.32%	42	8.08%	
**Mean follow-up** (years, mean ± SD)	5.89 ± 2.78		5.26 ± 2.44		<0.0001
**Median (IQR) follow-up** (years)	5.39 (2.68–6.95)		4.87 (2.30–6.28)		<0.0001
**All-Cause Death**					0.1585
No	1529	75.32%	376	72.31%	
Yes	501	24.68%	144	27.69%	

PET–CT, positron emission tomography–computed tomography; AJCC, American Joint Committee on Cancer; CCI, Charlson comorbidity index; RT, radiotherapy; CCRT, concurrent chemoradiotherapy; SD, standard deviation; IQR, interquartile range; y, years.

**Table 2:** Cox-proportional-hazards regression analysis of the risk of all-cause death among propensity score–matched patients with cervical cancer receiving curative surgery.

	HR (95% CI)		*p* Value	aHR * (95% CI)		*p* Value
**Preoperative PET-CT** (ref. No)						
Yes	1.23	(1.02–1.48)	0.0314	1.30	(1.07–1.58)	0.0076
**Age** (ref. Age ≤ 40 y)						
40 y < Age ≤ 50 y	1.19	(0.92–1.54)	0.1774	0.93	(0.72–1.21)	0.6058
50 y < Age ≤ 60 y	1.39	(1.09–1.77)	0.0070	0.99	(0.77–1.27)	0.9445
Age > 60 y	1.83	(1.44–2.33)	<0.0001	1.20	(0.93–1.55)	0.1676
**CCI Score** (ref. = 0)						
≥1	1.68	(1.4–2.02)	<0.0001	1.37	(1.13–1.66)	0.0015
**Years of Diagnosis** (ref. = 2008–2010)						
2011–2014	0.64	(0.53–1.07)	0.1892	0.78	(0.64–1.06)	0.3203
2015–2018	0.66	(0.54–1.08)	0.1679	0.81	(0.64–1.09)	0.4267
**Differentiation** (ref = well–differentiated)						
II (moderately differentiated)	1.17	(0.82–1.67)	0.379	1.09	(0.90–1.99)	0.1751
III (poorly differentiated)	1.58	(0.91–2.25)	0.122	1.17	(0.85–2.41)	0.1067
IV (undifferentiated)	3.35	(0.92–5.86)	0.098	1.26	(0.91–3.49)	0.2276
**AJCC Clinical stages** (ref. = stage IB)						
IIA	1.44	(1.06–1.96)	0.0191	1.09	(1.04–1.50)	<0.0001
**Neoadjuvant/Adjuvant treatment** (ref = No neoadjuvant/adjuvant)						
Adjuvant RT	1.12	(0.86–2.35)	0.1708	1.03	(0.87–2.69)	0.1472
Neoadjuvant CCRT	1.16	(0.83–2.47)	0.2157	1.08	(0.84–2.28)	0.2902
Neoadjuvant CT	1.28	(0.93–3.13)	0.1453	1.14	(0.66–3.99)	0.6451
Adjuvant CCRT	1.35	(0.89–2.04)	0.1555	1.22	(0.88–2.31)	0.1751
**Surgical margin (ref = negative)**						
Positive	2.65	(2.25–3.12)	<0.0001	1.98	(1.66–2.36)	<0.0001
**Histological type** (ref = Squamous cell carcinoma)						
Adenocarcinoma	1.55	(1.30–1.84)	<0.0001	1.85	(1.54–2.22)	<0.0001
**Medical Center** (ref = No)						
Yes	0.85	(0.72–1.00)	0.0547	0.98	(0.91–1.33)	0.3397

PET–CT, positron emission tomography–computed tomography; HR, hazards ratio; aHR, adjusted hazard ratio; CI, confidence interval; AJCC, American Joint Committee on Cancer; CCI, Charlson comorbidity index; RT, radiotherapy; CCRT, concurrent chemoradiotherapy; y, years; ref, reference group. * All covariates mentioned in Table 2 were adjusted.

**Table 3:** The change of stage (clinical stage convert to pathologic stage) for PSM patients receiving thoracic surgery.

		Pathologic Stages									
		IB		IIA		IIB		III		IVA	
	Patient No.	n	(%)	n	(%)	n	(%)	n	(%)	n	(%)
**Clinical stage IB**											
PET	364	240	(65.93)	47	(12.91)	42	(11.54)	20	(5.49)	15	(4.12)
Non-PET	1421	1135	(79.87)	128	(9.00)	85	(5.98)	56	(3.94)	17	(1.20)
**Clinical stage IIA**											
PET	156	15	(9.63)	110	(70.51)	13	(8.33)	10	(6.41)	8	(5.13)
Non-PET	609	23	(3.78)	506	(83.09)	31	(5.09)	30	(4.93)	19	(3.12)

%: Consistency percentage of Clinical stage and pathologic stage through PET or non-PET staging.

## Data Availability

The datasets supporting the study conclusions are included in the manuscript. We used data from the National Health Insurance Research Database and Taiwan Cancer Registry database. The authors confirm that, for approved reasons, some access restrictions apply to the data underlying the findings. The data used in this study cannot be made available in the manuscript, the supplemental files, or in a public repository due to the Personal Information Protection Act executed by Taiwan’s government, starting in 2012. Requests for data can be sent as a formal proposal to obtain approval from the ethics review committee of the appropriate governmental department in Taiwan. Specifically, links regarding contact info for which data requests may be sent to are as follows: http://nhird.nhri.org.tw/en/Data_Subsets.html#S3 (accessed on 31 December 2013) and http://nhis.nhri.org.tw/point.html (accessed on 31 December 2013).

## Abbreviations

PET–CT	positron emission tomography–computed tomography
^18^FDG-PET–CT	18-fluorodeoxyglucose positron emission tomography–computed tomography
HR	hazard ratio
aHR	adjusted hazard ratio
CI	confidence interval
NCCN	National Comprehensive Cancer Network
RCT	randomized controlled trial
PSM	propensity score matching
HWDC	Health and Welfare Data Center
NHI	National Health Insurance
AJCC	American Joint Committee on Cancer
CCI	Charlson comorbidity index
OS	overall survival
ICD-10-CM	International Classification of Diseases, Tenth Revision, Clinical Modification
ICD-9-CM	International Classification of Diseases, Ninth Revision, Clinical Modification
RT	radiotherapy
CCRT	concurrent chemoradiotherapy
CT	computed tomography
MRI	magnetic resonance imaging
NHIRD	National Health Insurance Research Database
SCC	squamous cell carcinoma

## References

[1] Health Promotion Administration (2017). Taiwan Cancer Registry Annual Report.

[2] Sung H., Ferlay J., Siegel R.L., Laversanne M., Soerjomataram I., Jemal A., Bray F. (2021). Global Cancer Statistics 2020: GLOBOCAN Estimates of Incidence and Mortality Worldwide for 36 Cancers in 185 Countries. CA Cancer J. Clin..

[3] Bray F., Ferlay J., Soerjomataram I., Siegel R.L., Torre L.A., Jemal A. (2018). Global cancer statistics 2018: GLOBOCAN estimates of incidence and mortality worldwide for 36 cancers in 185 countries. CA Cancer J. Clin..

[4] Vistad I., Fossa S.D., Dahl A.A. (2006). A critical review of patient-rated quality of life studies of long-term survivors of cervical cancer. Gynecol. Oncol..

[5] Kaneyasu Y., Fujiwara H., Nishimura T., Sakurai H., Kazumoto T., Ikushima H., Uno T., Tokumaru S., Harima Y., Gomi H. (2021). A multi-institutional survey of the quality of life after treatment for uterine cervical cancer: A comparison between radical radiotherapy and surgery in Japan. J. Radiat. Res..

[6] Landoni F., Maneo A., Colombo A., Placa F., Milani R., Perego P., Favini G., Ferri L., Mangioni C. (1997). Randomised study of radical surgery versus radiotherapy for stage Ib-IIa cervical cancer. Lancet.

[7] Morice P., Rouanet P., Rey A., Romestaing P., Houvenaeghel G., Boulanger J.C., Leveque J., Cowen D., Mathevet P., Malhaire J.P. (2012). Results of the GYNECO 02 study, an FNCLCC phase III trial comparing hysterectomy with no hysterectomy in patients with a (clinical and radiological) complete response after chemoradiation therapy for stage IB2 or II cervical cancer. Oncologist.

[8] Darus C.J., Callahan M.B., Nguyen Q.N., Pastore L.M., Schneider B.F., Rice L.W., Jazaeri A.A. (2008). Chemoradiation with and without adjuvant extrafascial hysterectomy for IB2 cervical carcinoma. Int. J. Gynecol. Cancer.

[9] NCCN (2021). Clinical Practice Guidelines in Oncology: Cervical Cancer.

[10] Signorelli M., Guerra L., Montanelli L., Crivellaro C., Buda A., Dell′Anna T., Picchio M., Milani R., Fruscio R., Messa C. (2011). Preoperative staging of cervical cancer: Is 18-FDG-PET/CT really effective in patients with early stage disease?. Gynecol. Oncol..

[11] Driscoll D.O., Halpenny D., Johnston C., Sheehy N., Keogan M. (2015). 18F-FDG-PET/CT is of limited value in primary staging of early stage cervical cancer. Abdom. Imaging.

[12] Lv K., Guo H.M., Lu Y.J., Wu Z.X., Zhang K., Han J.K. (2014). Role of 18F-FDG PET/CT in detecting pelvic lymph-node metastases in patients with early-stage uterine cervical cancer: Comparison with MRI findings. Nucl. Med. Commun..

[13] Zhang J., Lu C.Y., Chen H.M., Wu S.Y. (2021). Neoadjuvant Chemotherapy or Endocrine Therapy for Invasive Ductal Carcinoma of the Breast with High Hormone Receptor Positivity and Human Epidermal Growth Factor Receptor 2 Negativity. JAMA Netw. Open.

[14] Zhang Z., Kim H.J., Lonjon G., Zhu Y., written on behalf of AME Big-Data Clinical Trial Collaborative Group (2019). Balance diagnostics after propensity score matching. Ann. Transl. Med..

[15] Austin P.C. (2011). Optimal caliper widths for propensity-score matching when estimating differences in means and differences in proportions in observational studies. Pharm. Stat..

[16] Yamashita H., Okuma K., Kawana K., Nakagawa S., Oda K., Yano T., Kobayashi S., Wakui R., Ohtomo K., Nakagawa K. (2010). Comparison between conventional surgery plus postoperative adjuvant radiotherapy and concurrent chemoradiation for FIGO stage IIB cervical carcinoma: A retrospective study. Am. J. Clin. Oncol..

[17] Kau Y.C., Liu F.C., Kuo C.F., Huang H.J., Li A.H., Hsieh M.Y., Yu H.P. (2019). Trend and survival outcome in Taiwan cervical cancer patients: A population-based study. Medicine.

[18] Zhang J., Qin L., Chen H.M., Hsu H.C., Chuang C.C., Chen D., Wu S.Y. (2020). Outcome patterns of cervical adenocarcinoma and squamous cell carcinoma following curative surgery: Before and after propensity score matching analysis of a cohort study. Am. J. Cancer Res..

[19] Olawaiye A.B., Baker T.P., Washington M.K., Mutch D.G. (2021). The new (Version 9) American Joint Committee on Cancer tumor, node, metastasis staging for cervical cancer. CA Cancer J. Clin..

[20] Peters W.A., Liu P.Y., Barrett R.J., Stock R.J., Monk B.J., Berek J.S., Souhami L., Grigsby P., Gordon W., Alberts D.S. (2000). Concurrent chemotherapy and pelvic radiation therapy compared with pelvic radiation therapy alone as adjuvant therapy after radical surgery in high-risk early-stage cancer of the cervix. J. Clin. Oncol. Off. J. Am. Soc. Clin. Oncol..

[21] Monk B.J., Wang J., Im S., Stock R.J., Peters W.A., Liu P.Y., Barrett R.J., Berek J.S., Souhami L., Grigsby P.W. (2005). Rethinking the use of radiation and chemotherapy after radical hysterectomy: A clinical-pathologic analysis of a Gynecologic Oncology Group/Southwest Oncology Group/Radiation Therapy Oncology Group trial. Gynecol. Oncol..

[22] Zhang J., Sun M., Li N., Miao M., Yang Y., Hsu H.C., Chen H.M., Wu S.Y. (2021). Contemporary external beam radiotherapy boost or high dose-rate brachytherapy boost for cervical cancer: A propensity-score-matched, nationwide, population-based cohort study. Am. J. Cancer Res..

[23] Zhang J., Qin L., Chen H.M., Hsu H.C., Chuang C.C., Chen D., Wu S.Y. (2020). Overall survival, locoregional recurrence, and distant metastasis of definitive concurrent chemoradiotherapy for cervical squamous cell carcinoma and adenocarcinoma: Before and after propensity score matching analysis of a cohort study. Am. J. Cancer Res..

[24] Wu S.-Y., Huang E.-Y., Lin H. (2019). Optimal Treatments for Cervical Adenocarcinoma. Am. J. Cancer Res..

[25] Deaton A., Cartwright N. (2018). Understanding and misunderstanding randomized controlled trials. Soc. Sci. Med..

[26] Austin P.C. (2011). An Introduction to Propensity Score Methods for Reducing the Effects of Confounding in Observational Studies. Multivar. Behav. Res..

